# Computed CD4 percentage as a low-cost method for determining pediatric antiretroviral treatment eligibility

**DOI:** 10.1186/1471-2334-8-31

**Published:** 2008-03-06

**Authors:** Steven FJ Callens, Faustin Kitetele, Jean Lusiama, Nicole Shabani, Samuel Edidi, Robert Colebunders, Frieda Behets, Annelies Van Rie

**Affiliations:** 1 Department of Epidemiology, School of Public Health, University of North Carolina at Chapel Hill, USA; 2 School of Public Health, University of Kinshasa, Democratic Republic of the Congo; 3 National AIDS Reference Laboratory, Kinshasa, Democratic Republic of the Congo; 4 Institute of Tropical Medicine, Antwerp, Belgium and University of Antwerp, Belgium

## Abstract

**Background:**

The performance of the WHO recommendations for pediatric antiretroviral treatment (ART) in resource poor settings is insufficiently documented in routine care.

**Methods:**

We compared clinical and immunological criteria in 366 children aged 0 to 12 years in Kinshasa and evaluated a simple computation to estimate CD4 percent, based on CD4 count, total white blood cell count and percentage lymphocytes. Kappa (κ) statistic was used to evaluate eligibility criteria and linear regression to determine trends of CD4 percent, count and total lymphocyte count (TLC).

**Results:**

Agreement between clinical and immunological eligibility criteria was poor (κ = 0.26). One third of children clinically eligible for ART were ineligible using immunological criteria; one third of children immunologically eligible were ineligible using clinical criteria. Among children presenting in WHO stage I or II, 54 (32%) were eligible according to immunological criteria. Agreement with CD4 percent was poor for TLC (κ = 0.04), fair for total CD4 count (κ = 0.39) and substantial for CD4 percent computational estimate (κ = 0.71). Among 5 to 12 years old children, total CD4 count was higher in younger age groups (-32 cells/mm^3 ^per year older), CD4 percent was similar across age groups.

**Conclusion:**

Age-specific thresholds for CD4 percent optimally determine pediatric ART eligibility. The use of CD4 percent computational estimate may increase ART access in settings with limited access to CD4 percent assays.

## Background

To facilitate the rapid scale-up of pediatric antiretroviral treatment (ART), the World Health Organization (WHO) published revised pediatric ART guidelines for resource poor settings in 2006 [1]. The ART eligibility criteria for infants and children rely on clinical and/or immunological thresholds and aim to identify those children with poor prognosis if ART initiation is delayed.

The WHO clinical staging system has been widely used in resource-limited countries, particularly in the African Region, and has proved pragmatic and useful in facilities at the primary care and referral level [1]. An evaluation of the guidelines in Zambia showed 20-fold higher mortality rates in children with WHO clinical stage IV compared to stages I and II, confirming the association of clinical stage with prognosis [2].

Similarly, WHO immunological thresholds for total CD4^+ ^T lymphocyte count (CD4 count) and CD4 percent (Table 1) are associated with prognosis, and correspond to a 5% annual mortality risk in US and European children aged one year or older [3]. An identical level of immunosuppression in children in resource poor settings may however not have the same prognostic value due to differences in genetic and environmental factors such as opportunistic infections or malnutrition [4,5]. For example, CD4 percent and CD4 count in healthy Kenyan children was markedly lower compared to children from Europe, USA and West Africa [6]. It is therefore uncertain whether the use of thresholds established through longitudinal studies in the US and Europe can be extrapolated to other regions in the world.

**Table 1:** Age-specific ART eligibility criteria for children in resource poor settings. Source: World Health Organization: Antiretroviral therapy of HIV infection in infants and children in resource-limited settings: towards universal access. Recommendations for a public health approach, 2006.

**Immunological marker**	**Age in months**			
	**≤ 11**	**12 – 35**	**36 – 59**	**≥ 60**
CD4 percent*	**<**25%	**<**20%	**<**15%	**<**15%
CD4 count (cells/mm^3^)*	**<**1500	**<**750	**<**350	**<**200
TLC (cells/mm^3^)^†^	**<**4000	**<**3000	**<**2500	**<**2000

* valid for children aged 0 to 12 years; ^† ^valid for children aged 0 to 8 years

We evaluated the impact of the 2006 WHO pediatric ART eligibility criteria on ART initiation decision making in a pediatric HIV care program in Kinshasa, Democratic Republic of Congo (DRC) and propose a lower cost alternative for the expensive CD4 percent assays.

## Methods

### Study setting

The family-centered "Sustainable AntiRetroviral Access (SARA)" Program at Kalembe Lembe pediatric hospital in Kinshasa started in November 2004 and was one of the first to provide free access to pediatric ART in the DRC. The program provides prophylaxis and treatment for opportunistic infections, psychosocial support, nutritional support, insecticide treated bed nets, as well as antiretroviral treatment, according to national guidelines. ART eligibility was defined based on the 2004 and 2005 WHO guidelines. Children presenting in WHO stage I or II were eligible for ART if CD4 percent was below 20% for children younger than 12 months, below 15% for children under the age of 12 years, or if CD4 count was below 200 cells/mm^3 ^for children older than 12 years. Children presenting at WHO stage III (prior to July 2005) or WHO stage III/IV (July 2005 to June 2006) were eligible irrespective of CD4 percent or CD4 count. First line ART consisted of stavudine, lamivudine and nevirapine for children weighing more than 15 kilograms and zidovudine, lamivudine and nevirapine for those weighing less.

### Study participants and data collection

HIV positive children and children exposed to HIV at birth were referred to the clinic from the greater Kinshasa area. All HIV infected children, aged 0 to 18 years that did not have alternative access to ART, are eligible for the SARA project. Data on children aged 0 to 12 years enrolled in the SARA program between November 30, 2004 and May 30, 2006 were analyzed for this study. Clinical and laboratory data collected at baseline were analyzed for all eligible children. Blood samples were collected in the morning and processed the same day at the National HIV Reference Laboratory in Kinshasa. CD4 percent and CD4 count were established using the single platform assay FACSCalibur (Becton Dickinson, Aalst, Belgium). Total lymphocyte count (TLC) was calculated based on WBC count and percent lymphocytes as determined by using the Absorbance Cytochemistry and Volume Technology 5-part differential (ACT 5 diff) hematology analyzer (Coulter Beckman, VWR international, France).

### Data Analysis

For the purpose of this analysis, we evaluated pediatric ART eligibility using clinical criteria and age-specific immunological criteria recommended in the 2006 WHO guidelines for pediatric ART in resource poor settings (Table 1) [1].

In addition to the CD4 percentage obtained by gold standard flow cytometry, i.e. FACSCalibur assay, we calculated the CD4 percent using the following formula: computed CD4 percent = absolute CD4 count/(WBC count × percentage lymphocytes). The CD4 count used in this computation was obtained by flow cytometry.

Fisher's exact test was used to compare proportions and Kappa (κ) coefficient to assess agreement between ART eligibility criteria. The κ coefficient was interpreted as follows: < 0.20: poor agreement, 0.21 – 0.40: fair agreement, 0.41 – 0.60: moderate agreement, 0.61 – 0.80: substantial agreement, and > 0.80: good agreement [7]. Linear regression was used to determine trends of CD4 percent, CD4 count and TLC across age groups and correlation between CD4 percent measured by flow cytometry and CD4 percent estimate derived through computation. Weight for age z-score was determined using the 1990 British Grow Charts references [8]. Malnutrition was defined as a weight for age z-score of less then minus two. Analyses were performed using STATA version 9.0 (STATA cooperation, Texas, USA).

### Ethical approval

All parents of patients signed informed consent for participation, children older than 12 years assented and children older than 8 years received an informational sheet about the study before enrollment. The SARA program was approved by the Institutional Review Boards of the University of North Carolina at Chapel Hill, USA, and the Kinshasa School of Public Health, DRC (IRB reference number 04-MED-168).

## Results

A total of 399 HIV infected, ART-naïve children aged 0 to 12 years, were enrolled in the SARA program between November 2004 and June 2006. Thirty-three children were excluded from this analysis because of missing CD4 percent, CD4 count or TLC results at time of enrollment; leaving 366 (91.7%) children for analysis. The median age at baseline was 5 years (Interquartile range (IQR): 1.7 to 8.0 years); the median CD4 percent, CD4 count and TLC by age group are presented in Table 2.

**Table 2:** Median (Interquartile range, IQR) CD4 percent, CD4 count obtained by flow cytometry and total lymphocyte count by age group.

**Age**	**n**	**CD4 percent**	**CD4 count (cells/mm^3^)**	**Total Lymphocyte Count (cells/mm^3^)**
< 12 m	53	31 (21 to 35)	2144 (1716 to 2822)	6232 (4752 to 9112)
12–35 m	68	15 (12 to 22)	1030 (600 to 1459)	5839 (3650 to 8582)
36–59 m	60	13 (9 to 22)	600 (282 to 867)	3619 (2528 to 4925)
5–8 years	91	17 (9 to 25)	470 (329 to 818)	2976 (1875 to 4100)
> 8 years	95	17 (9 to 22)	415 (189 to 626)	2200 (1485 to 3162)

### Comparison of clinical and immunological ART eligibility criteria

At baseline, 196 (53.5%) of 366 children were eligible for ART on clinical grounds (WHO clinical stage III or IV) and 202 (55.2%) were eligible based on any of the age-specific immunological criteria (CD4 percent, CD4 count or TLC). Agreement between clinical and immunological eligibility criteria was poor (κ = 0.26). One third (32.6%, 64/196) of children eligible by clinical criteria were not eligible according to immunological criteria and one third (34.7%, 70/202) of children eligible on immunological criteria were not eligible by clinical criteria. Agreement between clinical and individual immunological criteria was poor to fair, with κ coefficients of 0.29 for CD4 percent, 0.20 for CD4 count and 0.08 for TLC.

### Evaluation of immunological criteria

Among children presenting with clinical stage I and II (i.e. ineligible for ART based on clinical criteria), 54/170 (31.8%) were ART eligible by age-specific CD4 percent, 24/170 (14.1%) were ART eligible by age-specific CD4 count and 20/125 (16%) by TLC criteria. Overall, the agreement between CD4 percent and CD4 count thresholds was fair (κ = 0.39), the agreement between CD4 percent and TLC was poor (κ = 0.04) (Table 3). Similar poor to fair agreements were observed across age groups (Table 3). Among children presenting with clinical stage I and II and eligible using CD4 percent thresholds measured by flow cytometry, only 7/38 (18.4%) children age 0 to 8 years would have been eligible using the TLC threshold and one in three (20/54 or 37.0%) children aged 0 to 12 years would have been eligible using CD4 count.

**Table 3:** Agreement (κ coefficient) using CD4 count obtained by flow cytometry, CD4 percent by computational estimate, and total lymphocyte count (TLC) compared to CD4 percent obtained by flow cytometry for children not eligible for ART on clinical grounds, i.e. WHO stage I or II

**Age group**	**n**	**CD4 count**	**CD4 percent computational estimate**	**TLC**
Overall	170/125*	77.6% (0.39)	87.6% (0.71)	64.8% (0.04)*
≤ 11 months	41	78.0% (0.19)	87.8% (0.63)	80.5% (0.32)
12 to 35 months	22	68.2% (0.32)	95.4% (0.91)	54.5% (0.02)
36 to 59 months	21	71.4% (0.25)	71.4% (0.35)	47.6% (-0.32)
60 months to 96 months	41	85.4% (0.58)	92.7% (0.82)	63.4% (0.04)
≥ 96 months	45	77.8% (0.45)	86.7% (0.71)	N/A†

* 125 children aged between 0 and 8 years; † Total lymphocyte count (TLC) is not valid in children older than 8 years (n = 45)

### Evaluation of the CD4 percent computational estimate

**Figure 1:**
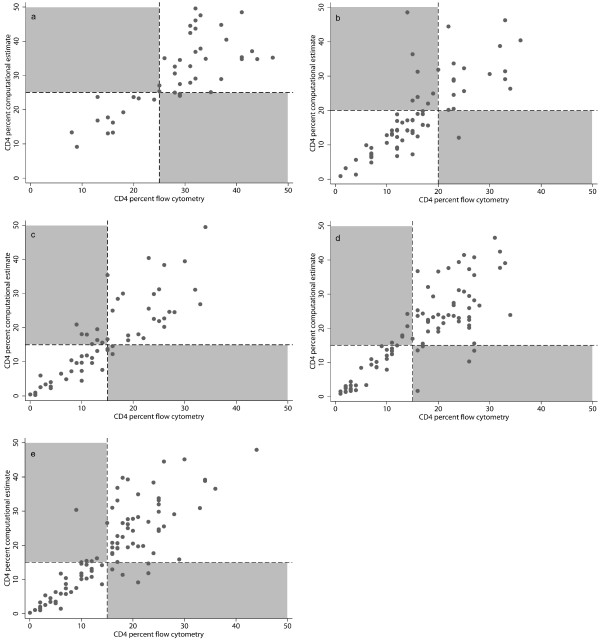
**CD4 percent obtained by flow cytometry and computational estimate by age group: (A) 0 to 11 months, (B) 12 to 35 months, (C) 36 to 59 months, (D) 60 to 95 months (E) more than 95 months.** Grey areas indicate children misclassified using CD4 percent computational estimate compared to CD4 percent obtained by flow cytometry.

The CD4 percent computational estimate was deemed biologically plausible in the majority (99%, n = 362) of children. In the remaining 1% (n = 4), the calculated CD4 percent was higher than 70%, a value rarely, if ever, observed in vivo, and possibly due to discrepant lymphocyte count obtained by staining and flow cytometry. Excluding the four values deemed implausible, the difference between the computational estimates and CD4 percent obtained by flow cytometry was largest for children with relatively high CD4 percent, and increased by 1.15% (95%CI 1.11 to 1.18, p < 0.01, r^2 ^= 0.91) for every percent increase in CD4 percent obtained by flow cytometry (Figure 1).

Among children presenting in clinical stage I or II, a similar proportion of children was eligible using the two different CD4 percent methods: 52 (31.3%) using CD4 percent obtained by flow cytometry and 53 (31.9%) using CD4 percent computational estimate (p = 0.9). The agreement between results of computational estimates, including those values deemed implausible, and CD4 percent obtained by flow cytometry was good (κ = 0.71) except for children age 35 to 59 months (Table 3).

In asymptomatic children, combining the CD4 computational estimate and CD4 count obtained with flow cytometry would identify 2 extra children eligible for ART treatment.

### Cross-sectional baseline CD4 count, CD4 percent and TLC by age

**Figure 2:**
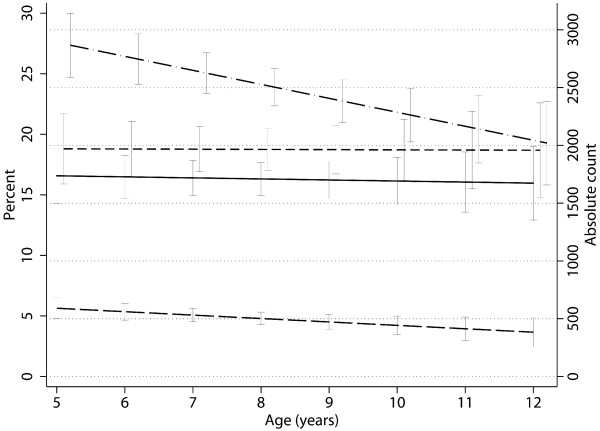
**Baseline total CD4 count and total lymphocyte count (TLC) decrease with increasing age (p < 0.01). **CD4 percent obtained by flow cytometryandcomputational estimate are stable with increasing age (p > 0.05). Linear prediction (and 95%CI) of CD4 percent obtained by flow cytometry (solid line), CD4 percent computational estimate (dashed line), total CD4 count (long dash line) and TLC (long dash – dotted line) in children aged 5 to 12 years.

Among children aged 5 to 12 years (n = 186), baseline CD4 count was significantly higher in younger children (-32 cells/mm^3 ^per year, 95%CI -58 to -5, p = 0.02). Similarly, a decrease in TLC was noted with increase in age (-198 cells/mm^3 ^per year, 95%CI -335 to -61, p < 0.01). In contrast, CD4 percent obtained by flow cytometry at (-0.8% per year (95%CI -0.73 to 0.57, p = 0.8) and computational estimate percent (-0.01%, 95%CI -0.97 to 0.77, p = 0.9) remained stable (Figure 2).

## Discussion

### Performance of the clinical criteria

Similar to experiences in Uganda and Haiti, approximately half of the children in our DRC cohort were eligible for ART at first presentation, likely due to late care-seeking and the recent introduction of free ART in these settings [9,10].

The agreement between the WHO clinical stages and immunological thresholds for ART eligibility was fair for CD4 percent but poor for total CD4 count and TLC. Approximately one third of children eligible for ART using immunological criteria were not eligible using clinical criteria. ART eligibility decision making on clinical grounds only would thus delay ART initiation in these children until they become symptomatic. Delay in ART initiation could lead to death in young infants who progress rapidly. Delayed ART initiation also impacts negatively immunological recovery following ART initiation, as patients with a low CD4 lymphocyte nadir have a slower increases in CD4 count, prolonging the period at risk of opportunistic infections [11]. Furthermore, when ART is started in children with less than 5% CD4 percent, levels of CD4 rarely reach normal levels [12].

Up to 30% of symptomatic children (WHO clinical stage III or IV) were not eligible for ART using immunological criteria. Children in Sub Saharan Africa are exposed to more infectious agents compared to children in industrialized countries. The high incidence of tuberculosis, bacterial dysentery, recurrent pneumonia and malnutrition may have resulted in a high proportion of children classified in WHO stages III/IV despite a relatively good immune system. Consequently, some children classified as clinical WHO stage III/IV could start ART "too early", if their symptoms are not the consequence of immunosuppression, but rather the result of environmental exposure. This was also observed in Tanzania by Johnson et al, who reported a sensitivity of 72% and a specificity of 56% of WHO clinical stage III/IV to detect severe immunosuppression in HIV positive children and has also been observed in adult populations [13,14]. In Cambodia, the 2003 WHO clinical criteria had a sensitivity of 96%, but an equally low specificity of 57% to identify patients in need of ART [14].

Because CD4 count and percentage only assess the level of immune suppression in a quantitative manner, not qualitatively, it would be imprudent to delay ART in children eligible based on clinical criteria. Results of the 3Cs4Kids a meta-analysis untreated HIV infected children in resource poor settings, showed that malnutrition, hemoglobin and WHO stage III or IV disease, are associated with high mortality independent of CD4 percentage or count, underscoring that ART should not be delayed in children with these conditions [15].

### Performance of the immunological criteria

Understanding the correlations between CD4 count, TLC, and CD4 percent is important in settings where access to the gold standard CD4 percent assay is limited. In our study, agreement between CD4 percent and total CD4 count or TLC was poor. Among children presenting in clinical stage I and II, only one third of children eligible according to CD4 percent criteria were eligible using total CD4 count, and only one in five children eligible based on CD4 percent were eligible according to TLC criteria. The CD4 percent computational estimate correlated better with the CD4 percent obtained by flow cytometry. More than 80% of ART children eligible according to the CD4 percent obtained by flow cytometry were also eligible using CD4 percent computational estimate. Use of the CD4 percent computational estimate could thus represent a substantial improvement compared to CD4 count. Moreover, it is an easy method, which can be implemented with limited extra cost beyond the cost of CD4 count, i.e. the cost to determine the total white blood cell count and lymphocyte percentage. Furthermore, the use of the CD4 percent computational estimate could facilitate decentralization of pediatric ART in centers without the access to flow cytometry, which may result in decreased indirect costs such as time off work and transportation cost.

Because a smaller proportion of children is eligible according to CD4 count compared to CD4 percent, relying on CD4 count for ART eligibility screening could delay ART initiation in up to 30% of children, exposing them to prolonged risk of acquiring opportunistic infections, disease progression or death. As the current WHO criteria are based on US en European data, the underestimation of children eligible for ART based on immunological criteria may be even more pronounced in sub-Saharan African settings, as genetic and environmental factors, which may influence immunity, are currently not taken into account in the WHO classification [15,16].

### Decline of CD4 count in children older than 5 years

WHO recommends the use of total CD4 count for ART eligibility screening in children older than 5 years. Similar to findings in Ugandan and Kenyan HIV negative children [5,6], CD4 count in HIV infected children in our study continued to decrease between the ages of 5 and 12 years. In contrast, both the CD4 percents measured by flow cytometry and the computational estimates were stable across age groups. Using CD4 count alone from the age of 5 might thus underestimate the number of children eligible for ART.

### Study Limitations

Several limitations to this study should be noted. To calculate the CD4 percent using the simple formula, we used the CD4 count as determined by flow cytometry, and although the lymphocytes were determined using an automated complete blood count (CBC) method using staining methods and not flow cytometry, the technique used is also automated and may be too costly in many settings. While others have demonstrated that low cost CD4 assays correlate well with the FACSCalibur assay [17,18], the use of the CD4 count obtained by flow cytometry may have resulted in an overestimation of the correlation of the CD4 percent computational estimates with the CD4 percent measured by flow cytometry. Prior to recommending the use of this formula in resource poor settings, the validity of this computational approach should therefore be evaluated using WBC count, percentage lymphocytes and CD4 count obtained through low cost (non flow-based) assays. Second, the finding that baseline CD4 count and TLC decreased with increasing age was based on cross sectional data analysis and not on longitudinal follow up of children. The observed decrease could also be due to increased immunosuppression with increasing age. However, one would then also expect a decreasing CD4 percent with age, whereas our data demonstrated stable CD4 percent values across the 5–12 years age group. Lastly, the value of different ART eligibility criteria would ideally be determined by their correlation with risk of death or disease progression, as in cohort studies conducted in European and USA in the early stage of the AIDS epidemic. Clearly, in light of the effectiveness of and increasing access to ART, such cohort studies can no longer be performed. The 3Cs4Kids meta-analysis showed that, compared to children in Europe and the US, children in resource limited settings have a higher 12-month risk of death at any level of CD4 percentage or count. However, this study could not determine whether this increased risk was attributable to immunosuppression or other factors, such as the higher mortality seen in severely malnourished children [15]. In contrast, trials, such as the CHER and PREDICT trial, establishing the impact of early ART initiation in children, might generate useful data on optimal timing of ART [19,20].

## Conclusion

CD4 percent is the most appropriate immunological criterion to screen HIV infected children, aged 0 to 12 years, for ART eligibility. CD4 percent will identify the highest proportion of children at early stages of immunosuppression. CD4 count and TLC may be less appropriate as the proposed WHO thresholds for total CD4 count and TLC do not correspond well with CD4 percent, CD4 counts decrease with increasing age, and using total CD4 count or TLC underestimates the number of children eligible for ART. Where access to CD4 percent assays is not available, CD4 percent obtained with a simple formula based on total white blood count, lymphocyte percent and total CD4 count may improve the identification of children in need of treatment in resource poor settings.

## Competing interests

The author(s) declare that they have no competing interests.

## Authors' contributions

SC wrote the manuscript; FK and JL coordinated the SARA project, collected data and reviewed the manuscript; SE performed the laboratory assays and reviewed the manuscript; RC, FB and AVR provided scientific input in the discussion of the article. All authors read and approved the final manuscript.

## Pre-publication history

The pre-publication history for this paper can be accessed here:


